# Paravertebral Abscess Secondary to Vertebral Osteomyelitis in an Intravenous Drug User

**DOI:** 10.7759/cureus.40230

**Published:** 2023-06-10

**Authors:** Marianne Cortes, Taylor Mazzei, Anuj Khanna, Kira Fenton, Cristina Savu

**Affiliations:** 1 Osteopathic Medicine, Nova Southeastern University Dr. Kiran C. Patel College of Osteopathic Medicine, Davie, USA; 2 Internal Medicine, Broward Health Medical Center, Fort Lauderdale, USA

**Keywords:** staphylococcus sp, cardiothoracic surgery, infectious disease pathology, staphylococcus aureus, intravenous drug user, pyogenic osteomylitis, paravertebral abscess, vertebral osteomyelitis

## Abstract

Hematogenous pyogenic vertebral osteomyelitis (VO) is a rare and often fatal complication of osteomyelitis that can affect individuals with underlying medical conditions, hospital-acquired infections, and intravenous (IV) drug abuse. Pyogenic vertebral osteomyelitis can present with generalized back pain, pyrexia, motor weakness, and neurologic deficits. The enigmatic presentation of this condition often results in delays in diagnosis and an increase in mortality. This case report aims to bring awareness to complications of hematogenous pyogenic vertebral osteomyelitis as well as highlight the need for further studies in order to establish standardized treatment. In our report, we depict a case of complicated pyogenic VO that required pharmacological and surgical intervention.

## Introduction

Vertebral osteomyelitis (VO) is a rare infectious condition that predominantly affects adults through hematologically derived seeding. While its occurrence is uncommon, it has gained prevalence throughout recent years due to an increase in the aging population, hospital-acquired infections, and intravenous drug use [1]. *Staphylococcus aureus* (*S. aureus*)continues to be the most implicated organism with methicillin resistance becoming more common and leading to worse outcomes [1,2]. Complications of these gram-positive organisms include infectious seeding which eventually leads to paravertebral, epidural, or psoas abscesses [3,4]. Diagnosis of a paravertebral abscess secondary to VO is particularly infrequent, reported in as little as 1 in 100,000-250,000 of the general population in developing countries, with little epidemiological data on the overall prevalence in North America [3]. The abuse of intravenous (IV) drugs has, however, been correlated to a higher risk of abscess formation secondary to contaminated syringes and needles [5]. Patients with additional underlying medical conditions such as diabetes mellitus, coronary heart disease, immunosuppression, malignancy, or renal failure have also been documented to be at increased risk [4]. The sporadic nature of hematogenous pyogenic VO along with its ambiguous clinical presentation can lead to delays in diagnosis.

## Case presentation

**Figure 1:**
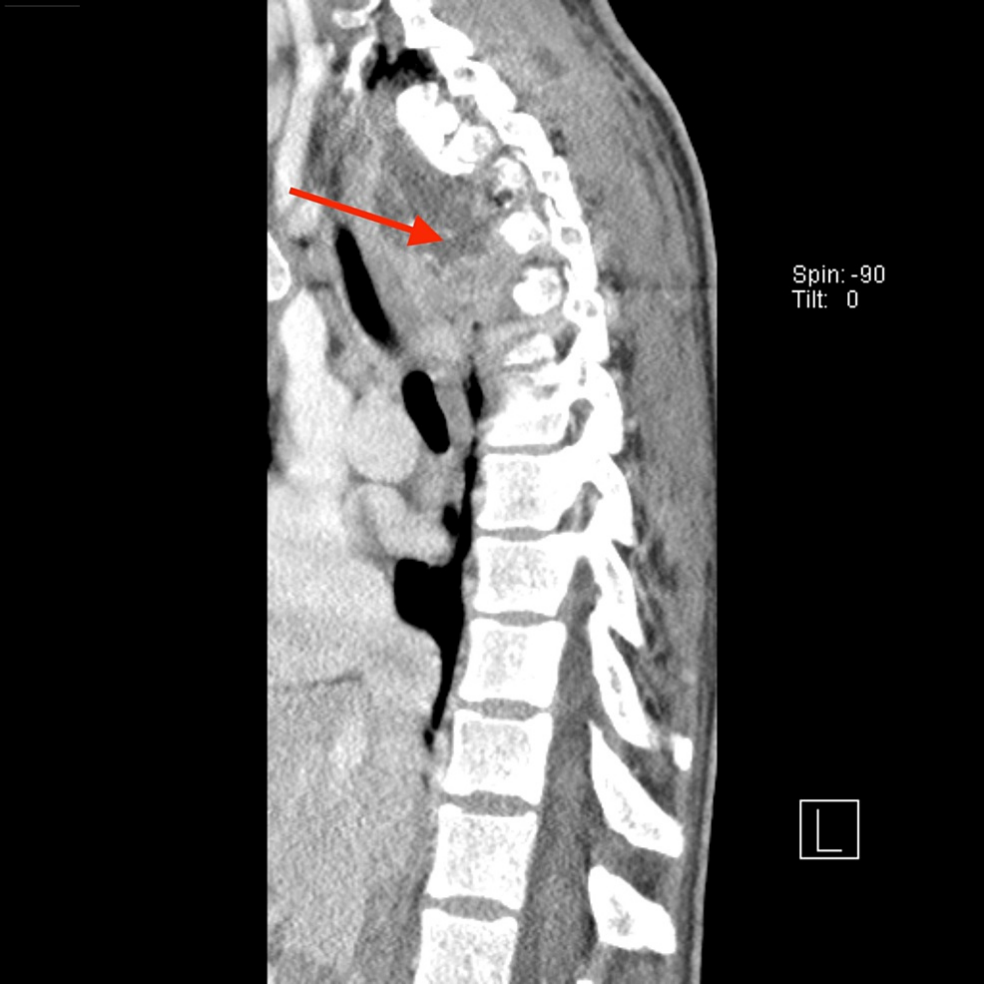
CT of the thoracic spine in a sagittal plane Sclerosis and erosive changes of T1, T2, and T4 can be noted. Associated with these bony abnormalities is a large paraspinal abscess that extends from the T1 level through the T5 level with measurements of approximately 7.5 cm in diameter with an anterior-posterior dimension of 3.3 cm, likely causing a mass effect upon the thoracic cord.

**Figure 2:**
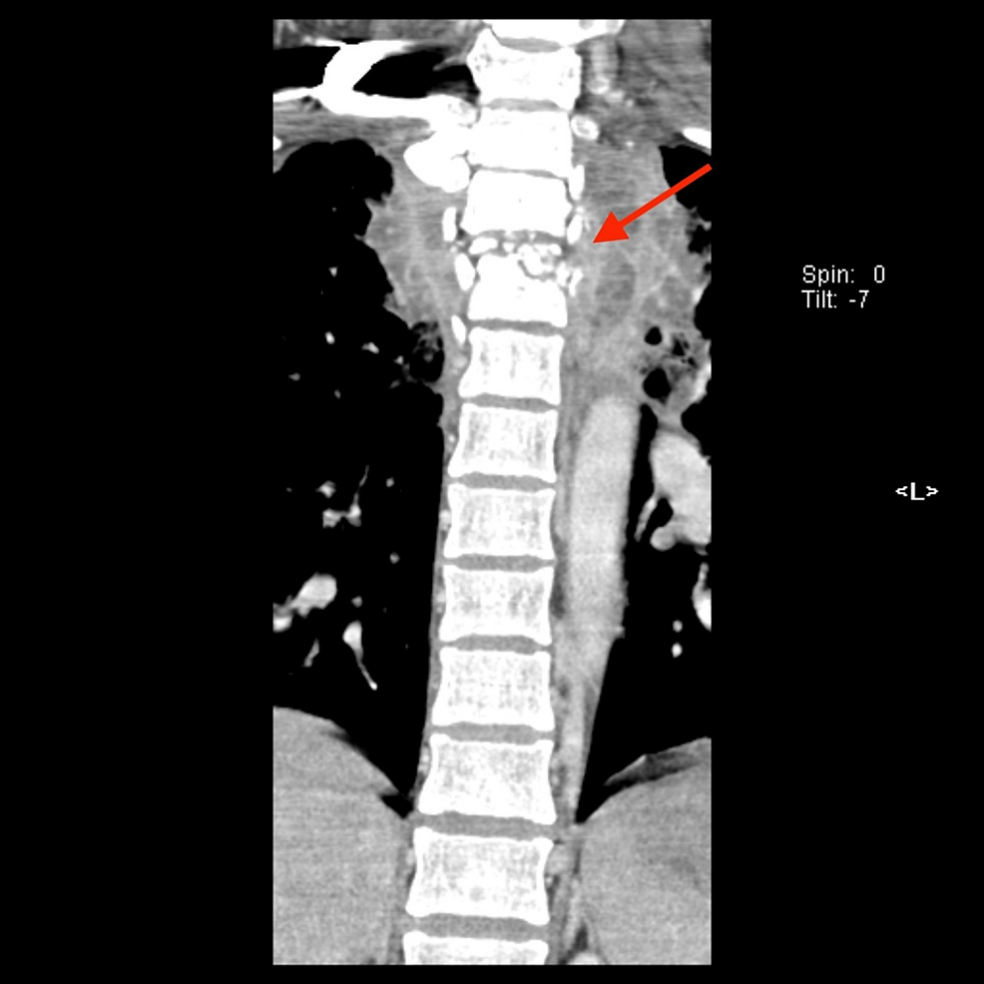
CT of the thoracic spine in a coronal plane Marked collapse and destruction of the T3 vertebral body.

A 37-year-old female with a past medical history of polysubstance abuse including IV drug use, chronic left forearm wound, hepatitis-C, iron deficiency anemia, and gunshot wound with a retained bullet in the right neck presented with severe back pain, difficulty ambulating, and incontinence for the past six weeks. Upon evaluation, the patient is responsive but very somnolent. It was reported that her last incidence of drug use was just prior to her arrival at the emergency department. Her vitals showed she had a low-grade fever of 100.1° F, was normotensive, and saturating well on the nasal cannula. Initial labs revealed a leukocytosis of 12,900/uL, elevated C-reactive protein (CRP) of 29.33 mg/dL, and erythrocyte sedimentation rate (ESR) of 131 mm/hr. CT imaging showed a large paravertebral abscess from T1-T4, mass effect with cord compression, and osteomyelitis (Figures 1, 2).

Aspiration of the paravertebral abscess and blood cultures were done which revealed a methicillin-susceptible *Staphylococcus aureus* (MSSA) infection. Transesophageal echocardiography (TEE) was negative for valvular vegetations. She was started on IV cefazolin with neurosurgical intervention involving posterior fixation of thoracic vertebrae with corpectomy on day 4 of admission. On day 7, bilateral video-assisted thoracic surgery (VATS) was performed for the paravertebral abscess with bilateral chest tube insertion after the procedure. She was subsequently admitted to the intensive care unit for further management and postoperative care.

During her postoperative time, the patient developed numerous pneumothorax upon attempted removal of chest tubes. Daily x-rays were performed to monitor further pneumothorax formation. The patient eventually had both chest tubes taken out without any complications or new pneumothorax formation. She continued to be stable for a few weeks with the exception of asymptomatic sinus bradycardia. She continued to work with physical therapy (PT) during this time until she was able to transfer herself into a wheelchair and properly ambulate with it. After a total of two months of inpatient postoperative care, she was discharged to a rehabilitation facility for further PT.

## Discussion

Hematogenous pyogenic VO mostly occurs in the setting of invasive procedures through transcutaneous infection of deep tissue by needles or catheters, surgery, blunt trauma, and hematogenous dissemination [3]. Through the modality of hematogenous seeding, the lumbar vertebral bodies are most commonly affected, followed by thoracic and cervical vertebrae [1]. Back pain, pyrexia, and muscular weakness are the most common presenting symptoms in cases of osteomyelitis with a paravertebral abscess which was also consistent in our case. More severe clinical symptoms include neurological deficits, altered sensation, motor function, bowel, and urinary dysfunction and ultimately paralysis [4]. Differential diagnoses in a febrile patient with back pain are broad and may include neoplastic, inflammatory, and infectious etiologies [3,4]. Lab results depicting increased leukocyte count, ESR, and CRP have also been reported in 98% and 100% of cases and can aid in ruling out other possible diagnoses [4]. Bone samples have been shown to have a higher overall diagnostic yield in comparison to blood cultures; however, the initial step of blood culturing may preclude the need for more invasive procedures [3,4,6]. Further diagnostic tools include MRI, which has been found to be ideal for identifying paravertebral abscess formation and high-signal-intensity marrow edema characteristic of vertebral destruction [3,4]. However, due to our patient’s retained bullet, we were unable to perform an MRI and instead used a CT for imaging. Careful inspection of imaging studies is recommended as the features of erosive osteochondrosis may mimic those of VO [4].

The 2015 Infectious Diseases Society of America (IDSA) has provided guidelines for the treatment of native vertebral osteomyelitis in adults which was applied in this case. Antibiotic treatment recommendations are aimed at medications effective against the most common pathogens of pyogenic VO such as *S. aureus*, Enterococcus, and* Pseudomonas aeruginosa *(*P. aeruginosa*) [6]. For patients expressing MSSA, the use of IV nafcillin, oxacillin, first-generation cephalosporins, or oral dicloxacillin are recommended. For those expressing methicillin-resistant *Staphylococcus aureus *(MRSA), vancomycin remains the gold standard [3]. Intravenous administration of these medications is suggested for a period of four to eight weeks up to three months depending on the organism involved [1,3]. Recent randomized trials are now proposing the possibility of an early switch to oral antibiotics such as fluoroquinolones as an effective treatment modality due to their excellent bioavailability [4]. While more literature is now being published, there continue to be limitations in the standardized management of complicated pyogenic VO. Due to our patient’s extensive bone destruction, antibiotics alone proved to be inadequate, and surgical intervention was deemed necessary to decompress and stabilize the spine. While surgical intervention is rarely performed due to its high variability, it may be indicated based on the degree of neurological deficit. Anterior approaches of debridement and strut grafting with delayed posterior fusions may be used to overcome spinal instability in patients with complicated VO [1].

## Conclusions

Through our case, we were able to present a rare condition along with the multiple treatment modalities needed to stabilize the patient. Timing is a crucial factor in VO in the presence of mass effect with cord compression, and management is necessary to avoid permanent disability. Standardized guidelines in the management of complicated osteomyelitis are lacking, making it difficult to manage these patients in a timely manner. We share our experience with a complicated case of VO in the hopes that there be a higher index of suspicion leading to efficient treatment.
